# First report on metagenomics and their predictive functional analysis of fermented bamboo shoot food of Tripura, North East India

**DOI:** 10.3389/fmicb.2023.1158411

**Published:** 2023-04-12

**Authors:** Rohit Das, Buddhiman Tamang, Ishfaq Nabi Najar, Nagendra Thakur, Krishnendu Mondal

**Affiliations:** ^1^Department of Microbiology, Sikkim University, Gangtok, India; ^2^Department of Microbiology, Vidyasagar University, Midnapore, India

**Keywords:** fermented bamboo shoot, *meloncana baccifera*, shotgun metagenomics, microbial community analysis, functional gene analysis

## Abstract

*Moiya pansung*, *mileye amileye*, *moiya koshak*, and *midukeye* are naturally fermented bamboo shoot foods of Tripura. The present study aimed to reveal the whole microbial community structure of naturally fermented *moiya pangsung, mileye amileye, moiya koshak*, and *midukeye* along with the prediction of microbial functional profiles by shotgun metagenomic sequence analysis. The metataxonomic profile of *moiya pangsung, mileye amileye, moiya koshak*, and *midukeye* samples showed different domains, viz., bacteria (97.70%) followed by the virus (0.76%), unclassified (0.09%), eukaryotes (1.46%) and archaea (0.05%). Overall, 49 phyla, 409 families, 841 genera, and 1,799 species were found in all the fermented bamboo shoot samples collected from different places of Tripura. Firmicutes was the most abundant phylum (89.28%) followed by Proteobacteria (5.13%), Bacteroidetes (4.38%), Actinobacteria (1.02%), and Fusobacteria (0.17%). *Lactiplantibacillus plantarum* was the most abundant species in *moiya pangsung, mileye amileye, moiya koshak*, and *midukeye* followed by *Lactococcus lactis, Levilactobacillus brevis, Leuconostoc mesenteroides, Weissella paramesenteroides, Leuconostoc kimchii, Pediococcus pentosaceus, Leuconostoc gasicomitatum*, and *Lacticaseibacillus casei.* A few phyla of fungus were found, viz., *Ascomycota, Basidiomycota*, and *Glomeromycota*, where *Ascomycota* was present in high abundance. Functional analysis of *moiya pangsung, mileye amileye, moiya koshak*, and *midukeye* metagenome revealed the genes for the synthesis and metabolism of a wide range of bioactive compounds including, various essential amino acids, and conjugated amino acids. The abundance profile and predictive analysis of fermented bamboo shoots revealed a huge plethora of essential microorganisms and KEGG analysis revealed genes for amino acid metabolism, pectin degradation, lipid metabolism, and many other essential pathways that can be essential for the improvement of nutritional and sensory qualities of the fermented bamboo shoot products.

## 1. Introduction

Bamboo is a long-living, robust, adaptable, and highly renewable species of grass that grows as woody stems and is primarily found in moist deciduous, semi-evergreen, tropical, subtropical, and temperate regions of forests (Tewari et al., 2019). There are more than 75 genera and 1,250 species of bamboo documented on a global scale. Bamboo shoots are a healthy vegetable that is frequently harvested, consumed, and sold by the rural tribes of North Eastern India (Singhal et al., 2018). Bamboo shoots have a great deal of promise as a food resource with a high protein and fiber content but with little fat, making them a wonderful source of nourishment. They include vital antioxidants and therapeutic ingredients that can help delay the onset of metabolic problems in addition to being a storehouse of nutritious materials (Hussain et al., 2020). The product becomes acidic and digestive due to the action of lactic acid bacteria at large and some yeast species (Ferreira and Mendes-Faia, 2020). The fermentation of bamboo shoots not only makes them very nutritious and increases their shelf life but also makes them pleasant in terms of flavor, aroma, texture, and appearance (Behera and Balaji, 2021).

Fermented bamboo shoots are commonly consumed in North East India and go by a variety of regional names, including *ekung* and *eup* in Arunachal Pradesh (Tamang and Tamang, 2009), *miyamikhri* and *khorisa* in Assam, *lung-siej*, *tuaithar*, and *tuairoi* in Mizoram, *soidon* and *soibum* in Manipur, *bastangapani* in Nagaland and *mesu* in Sikkim (Tamang et al., 2008). Bamboo shoots are a rich source of nutrients, powerful antioxidants, and healing compounds that can postpone the onset of certain metabolic diseases (Yu et al., 2021). Colossal diversity of microorganisms has been reported from different types of fermented bamboo shoots, including the species of bacteria. *Lactobacillus johnsonii, Lb. plantarum, Lb. brevis, Lb. fermentum, Lb. curvatus, Leuconostoc mesenteroides, Leuconostoc lactis, Pediococcus* sp*., Micrococcus* sp*., Bacillus* sp., and the yeasts species, viz., *Saccharomyces cerevisiae, Zygosaccharomyces rouxii, Yarrowia lipolytica*, and *candida* sp. (Tamang et al., 2012; Nongdam, 2015; Voidarou et al., 2021).

A total of 21 species of bamboo are reported in Tripura among which *muli bans* [*Meloncana. baccifera* (Roxb.) Kurz] is the most important bamboo of the state while other species contribute only 5–7% of total production. About 708 metric tonnes of young bamboo shoots are harvested from Tripura, of which *M. baccifera* constitutes about 80–85% of the total harvest (Banik, 2010). Different ethnic communities, viz., Chakma, Debbarma, and Uchoi use young tender shoots of *muli bans* to prepare fermented products like *moiya pangsung, mileye amileye, moiya koshak*, and *midukeye* (Uchoi et al., 2015). All the fermented samples collected from Tripura are still prepared at a small household level by the ethnic group using rudimentary tools, so the microbiota of fermented foods is very essential to be studied to better understand the dominating microbes responsible for fermentation as well as to know the impact on the health of the consumers. This fermented bamboo shoot is usually prepared by aged women of the community.

The microbiology of fermented bamboo shoots has been rarely studied through culture-independent techniques except in *tuaithar* of Mizoram (Deka et al., 2021), *khorisa* of Assam (Behera et al., 2020), *suansun* of China (Hu et al., 2021). Moreover, there are no culture-independent studies present on the fermented bamboo shoot products of Tripura.

Since *moiya pangsung, mileye amileye, moiya koshak*, and *midukeye* are traditionally prepared from the young bamboo shoot by natural fermentation, diverse species of bacteria and fungi present in the local environment may appear in the final product. Moreover, there is no report on the predictive functionalities of microbial genes present in this fermented bamboo shoot. Predictive functional profiles of microbial communities in fermented foods are an appropriate approach to annotating the predictive metabolic pathways in gene sequences of bacteria and fungi (Sun et al., 2022) using different pipelines of bioinformatics tools such as Metagenomic Rapid Annotations using Subsystems Technology (MG-RAST) (Keegan et al., 2016), and the Kyoto Encyclopedia of Genes and Genomes (KEGG) database (Kanehisa and Goto, 2000) for the bacterial gene. Hence, we aimed to study the bacterial and fungal community structure by shotgun sequencing tool during the natural fermentation of *moiya pangsung, mileye amileye, moiya koshak*, and *midukeye* of Tripura in India. We also aimed to predict the functional profiles of bacterial and fungal genes by using the bioinformatics pipelines, MG-RAST, during the natural fermentation of these fermented bamboo shoots.

## 2. Materials and methods

### 2.1. Sampling and site description

A total of 6 samples corresponding to four different types of fermented bamboo shoots were sampled in duplicates from different households and marketplaces in Tripura. A sample of *mileye amileye* was collected from the Panisagar district (PBMA) market (24.263307, 92.152439), 2 samples of *midukeye* were collected from the Dharmanagar household (DBMD) and Panisagar district (PBMD) (24.380504, 92.166556, and 24.269956, 92.150036, respectively), 2 samples of *moiya pangsung* were collected from Manubazar (MBMP) market place and Dharmanagar household (DBMP) (24.393275, 92.166073, and 23.074567, 91.645094, respectively) and 1 sample of *moiya koshak* was collected from Dharmanagar household (DBMK) (24.379511, 92.165093). All collected samples were kept in an ice box carrier and immediately transported within 24 h, where they were stored at −20°C for further analysis. Before fermentation, the sheaths of the young tender shoots were removed and then washed with water. *Moiya pangsung* is prepared from a top tender shoot and fermented for 2 days at room temperature in a closed container completely submerged in water (Supplementary Figure 1A), while *mileye amileye* is also fermented in a similar way but the middle portion of the bamboo shoot is used (Supplementary Figure 1B). Similarly, for *midukeye* and *moiya koshak*, the middle portion of the tender shoot is cut into pieces, then wrapped in banana leaves and left at room temperature for 2 days (Supplementary Figure 1C). These bamboo shoot products are sour and with a sweet and earthy after-taste. The type of samples and code name assigned to the samples are mentioned in Supplementary Table 1.

### 2.2. DNA extraction and sequencing

In a stomacher (400 Circulator, Seward, UK) ten grams of each sample were homogenized with 90 mL of sterile 0.1 M phosphate buffer saline (pH 6.4). The homogenate was filtered after homogenization, and the filtrate was utilized to extract genomic DNA using the Maggenome XpressDNA plant kit (Maggenome, USA) as per the manufacturer’s protocol. Using 0.8% agarose gel electrophoresis, the DNA samples were separated to check for deterioration and contaminants and to assess quality (voltage 100 V, period 40 min). A precise measurement of the total DNA mass concentration was obtained utilizing nanodrop (Eppendorf Biospectrometer@ basic, Germany), to confirm that the parameters for the library creation adhered to. Following the manufacturer’s instructions, total DNA was submitted to DNA library construction and amplification using the TruSeqTM DNA Sample Prep Kit (Illumina, United States) and the cBOT TruSeq PE Cluster Kit v3 (Illumina, United States) reagents. The TruSeq SBS Kit v3-HS (Illumina, United States) was used to sequence the DNA libraries utilizing the Illumina Novaseq 6,000 technology. According to the established Illumina methods, the sample DNA libraries were submitted to a paired-end (2 × 150 bp) sequencing platform. Fastq format was used to store the raw reads.

### 2.3. *De novo* metagenome assembly

High-quality filtered paired-end libraries of the samples of raw reads were assembled separately using MetaSPAdes assembler with default parameters (Nurk et al., 2017). The metagenomic data obtained from MetaSPAdes was run through the Galaxy platform^1^ (Afgan et al., 2018) using the default parameters.

### 2.4. Taxonomic classification and abundance

The putative microbial population reads from six samples were classified using Kraken2 against the reference database containing all Refseq bacterial, archaeal, fungal, and viral genomes with a 0.1 confidence threshold (Marcelino et al., 2020). After the classification by Kraken2, Bracken was used to re-estimate the different microbial abundances at taxa levels from species to phylum using a read length parameter of 150 (Terrón-Camero et al., 2022).

### 2.5. Predictive functionality

The gene prediction for protein-coding genes (ORFs) was predicted using Prodigal (Hyatt et al., 2010). ORFs shorter than 180 nucleotides were excluded from further analysis by default. tRNA genes were predicted with the ARAGORN program (Laslett and Canback, 2004), and Ribosomal RNA genes (5S, 5.8S, 16S, 18S, 23S, 28S) were identified and classified using rRNAFinder (Dong and Strous, 2019). All ORFs were searched against the evolutionary genealogy of genes, non-supervised Orthologous Groups (eggnog) database (Muller et al., 2009), for Clusters of Orthologous Groups/Non-supervised Orthologous Groups (COG/NOG) annotation (Galperin et al., 2018) and Kyoto Encyclopedia of Genes and Genomes (KEGG) database (Kanehisa and Goto, 2000) for KEGG ID annotation.

### 2.6. Statistical analysis

#### 2.6.1. Pooled sequences

Nucleotide diversity (pi) analysis and different indices of neutrality test based on sequence polymorphism in DnaSP software version 6 (Rozas et al., 2017) were performed to justify the pooling of DNA from each duplicate sample in terms of intra-sample (within the sample) diversity. Among the different indices of neutrality tests, Tajima’s D value and Fu’s Fs statistics were performed in the same software. To check the intra-sample diversity in terms of species level and functional profiles, one sample *T*-test was performed in IBM SPSS version 20 for all the samples. Statistical relations among the samples were performed using the Mann-Whitney test (Fong and Huang, 2019) in terms of species level and level 3 functional profiles in PAST version 4.0 software (Martino et al., 2019).

#### 2.6.2. Alpha and beta diversity indexes

Non-parametric Shannon index, Simpson’s index of diversity (1-D), Chao-1, and evenness were calculated using PAST software version 4.0. The Bray-Curtis index of beta diversity was also calculated using past version 4.0 and visualized *via* a Principal Coordinates Analysis (PCoA) plot (Martino et al., 2019).

#### 2.6.3. Functional profiling

The clustering pattern among the samples was checked through the PCoA plot using the level 3 sub-pathways. The PCoA plot was constructed after the log transformation of data and then visualized in the ClustVis web tool (Metsalu and Vilo, 2015). Non-parametric Spearman’s rank correlation was performed using IBM SPSS software version 20 and PAST version 4.0 (Martino et al., 2019) software to check the correlation between the microorganisms in samples and different levels (super-pathways) functional profiles. Heatmap was constructed *via* the ClustVis web tool to visualize the correlation profiles. Correlation between different bacteria and the amino acid profiles obtained from KEGG annotation was also performed.

## 3. Results

### 3.1. Pooled sequences

As mentioned earlier, each sample was collected in duplicates and was pooled on the basis of their geographical origin and we hypothesized that the samples from the same origin possess minimal diversity differences. Nucleotide diversity (pi) per site was 0.712 (PBMA), 0.713 (DBMD), 0.713 (PBMD), 0.733 (DBMK), 0.721 (DBMP), and 0.712 (MBMP), respectively. However, the theta values (nucleotide difference) found for each of the samples were 0.72, 0.73, 0.72, 0.72, and 0.73, respectively. First, we checked Tajima’s D value among the different indices of the neutrality test. Each sample showed a negative value for Tajima’s D, which were −0.21 (PBMA), −0.19 (DBMD), −0.19 (PBMD), −0.20 (DBMK), −0.21 (DBMP), and −0.19 (MBMP), respectively. The second neutrality test indices were Fu’s F_*S*_ statistics (FST); FST values found for all the samples were −5.30, −3.24, −3.24, −3.88, −5.21, and −3.90.

### 3.2. Taxonomic classification and abundance profiling

**FIGURE 1:**
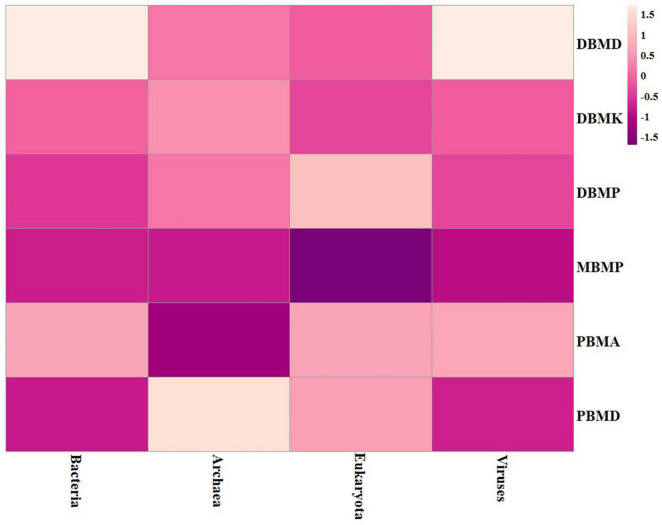
Heat map representation of major domain in *moiya pangsung, mileye amileye, moiya koshak*, and *midukeye*.

**FIGURE 2:**
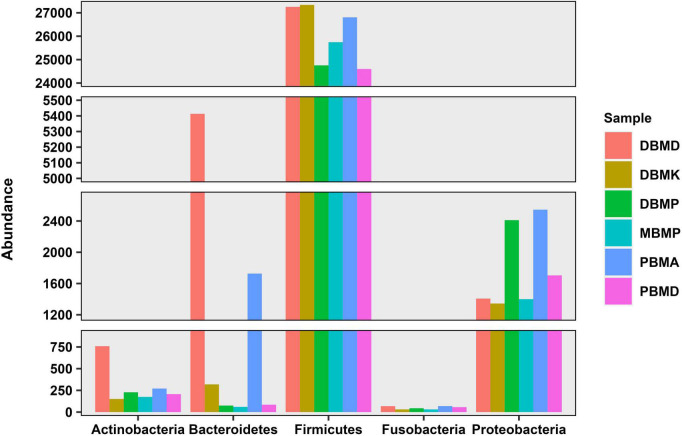
Bar plot for major phylum in bacteria.

**FIGURE 3:**
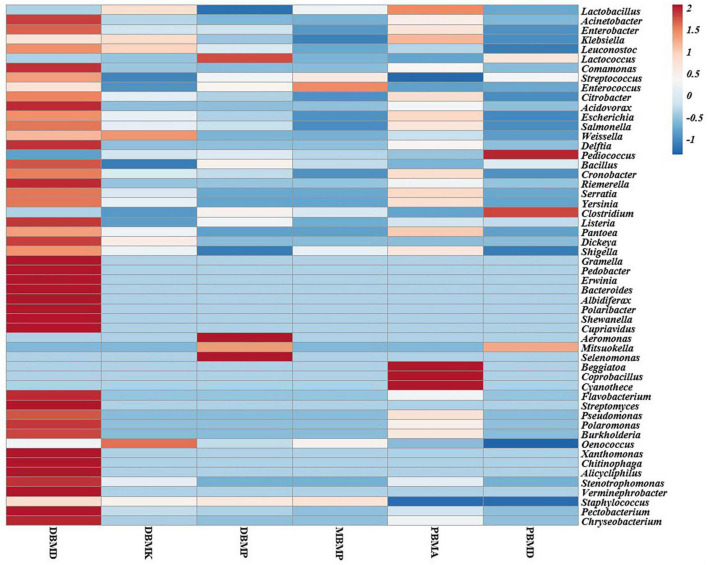
Heat map representation of major bacterial genera in *moiya pangsung, mileye amileye, moiya koshak*, and *midukeye*.

**FIGURE 4:**
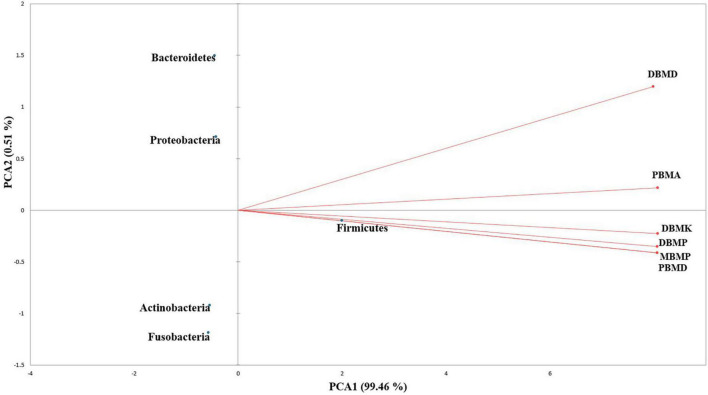
PCA biplot analysis of samples and microbial diversity at the genus level.

**FIGURE 5:**
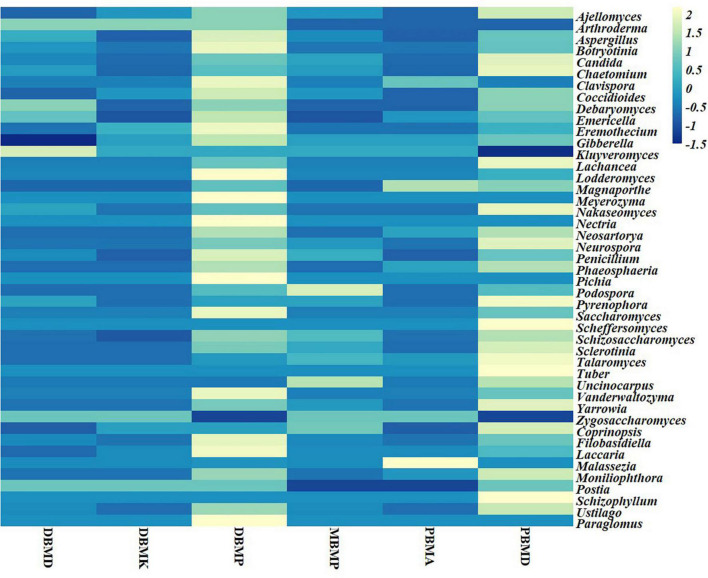
Heat map representation of major fungal genera communities in *moiya pangsung, mileye amileye, moiya koshak*, and *midukeye*.

A total of 1,570,915 reads were obtained from the samples with an average of 261,820 reads per sample. The average length of the reads was found 583 bp. The total number of bases recovered from the samples was 311,915,402 (DBMP), 285,708,731 (DBMD), 93,242,563 (MBMP), 71,456,540 (DBMD), 61,889,925 (PBMA), and 55,160,324 (DBMK), respectively. Shotgun metagenomic sequence analysis of *moiya pangsung, mileye amileye, moiya koshak*, and *midukeye* samples showed different domains, viz., bacteria, archaea, viruses, and eukaryotes. The total relative abundance was calculated in all the samples where bacteria were the most abundant domain (97.70%) followed by the virus (0.76%), unclassified (0.09%), eukaryotes (1.46%) and archaea (0.05%). Among samples, DBMD was seen to have the highest bacterial abundance (28.84%), PBMD had the highest archaeal abundance (24.46%), DBMP had the highest eukaryotic abundance of (0.41%), and DBMD had the highest viral abundance (31.34%) (Figure 1). A total of 49 phyla, 409 families, 841 genera, and 1,799 species were recorded after abundance analysis. At the bacterial phylum level, Firmicutes was the most abundant phylum (Figure 2). Across all the bacterial families, *Lactobacillaceae* was found the most abundant family followed by *Enterobacteriaceae* (Supplementary Table 2). *Lactobacillus was* found to be the most abundant genera of all, among them PBMA had the highest abundance (Figure 3). No cluster was found among the samples except PBMD and MBMP in PCoA analysis according to the abundance/distribution of bacterial genera from different samples (Figure 4). At the species level, *Lactiplantibacillus plantarum, Levilactobacillus brevis, Lactococcus lactis, Pediococcus pentosaceus, Leuconostoc mesenteroides*, and *Leuconostoc citreum* (Supplementary Figure 2), non-LAB, archaea, yeasts, filamentous moulds, other phages, microeukaryotes, and parasites were also detected in < 1% abundance from *moiya pangsung, mileye amileye, moiya koshak*, and *midukeye* samples. From the Eukaryota domain, 3 phyla of fungus were found *Ascomycota, Basidiomycota*, and *Glomeromycota*, where *Ascomycota* was present in abundance (Supplementary Figure 3A), *Saccharomycetaceae* was overall the most abundant family (Supplementary Figure 3B), *Schizosaccharomyces* was overall the most abundant fungal genera (Figure 5), and *Schizosaccharomyces pombe* was found to be the most abundant species on an overall basis (Supplementary Figure 3C).

### 3.3. Diversity indices

**FIGURE 6:**
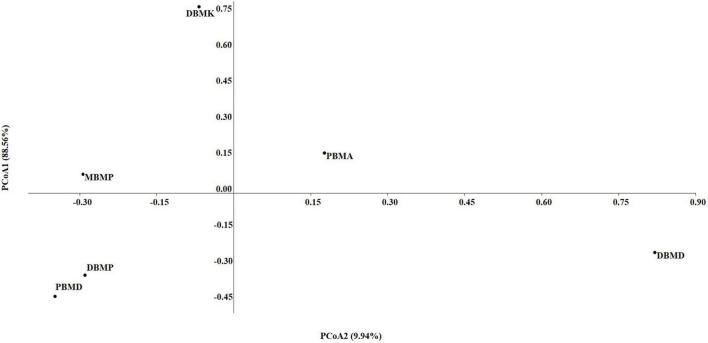
PCA plot for beta diversity analysis *via* Jaccard at the bacterial genus level.

**FIGURE 7:**
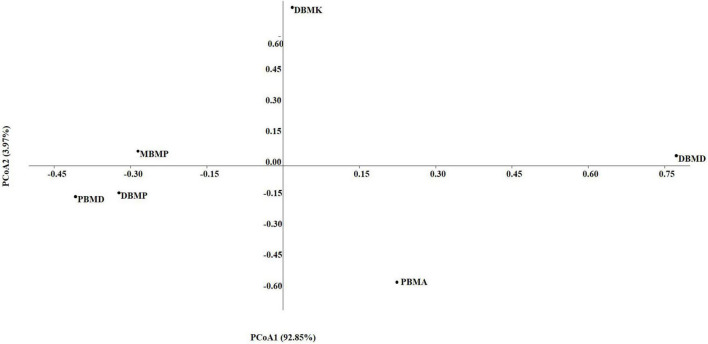
PCA plot for beta diversity analysis *via* Bray-Curtis at the bacterial genus level.

The non-parametric Shannon, Simpson, Chao-1, and Evenness indices were highest in the DBMD sample (Table 1). At the genus level, the beta diversity of DBMP and PBMD were completely different from other samples as visualized in principal component analysis obtained through Jaccard indices (Figure 6). Similarly, Bray-Curti’s beta diversity at the genus level also revealed DBMD and PBMD were completely different from the other 4 samples (Figure 7). The shared and unique species were visualized between 4 samples using a venn diagram elaborated that 61, 8, 1, and 85 species were unique to *moiya pangsung, moiya koshak, mileye amileye*, and *midukeye*, respectively, whereas 306 species were common in all the fermented products (Supplementary Figure 4).

**TABLE 1:** Alpha diversity indexes.

Sample	Simpson	Shannon	Chao-1	Evenness
DBMD	0.9232	3.009	49	0.4104
DBMK	0.8332	2.318	28	0.3626
DBMP	0.8957	2.285	23	0.4271
MBMP	0.7258	1.782	17	0.3495
PBMA	0.8698	2.626	38	0.3657
PBMD	0.7679	1.881	16	0.4102

### 3.4. Functional profile

**FIGURE 8:**
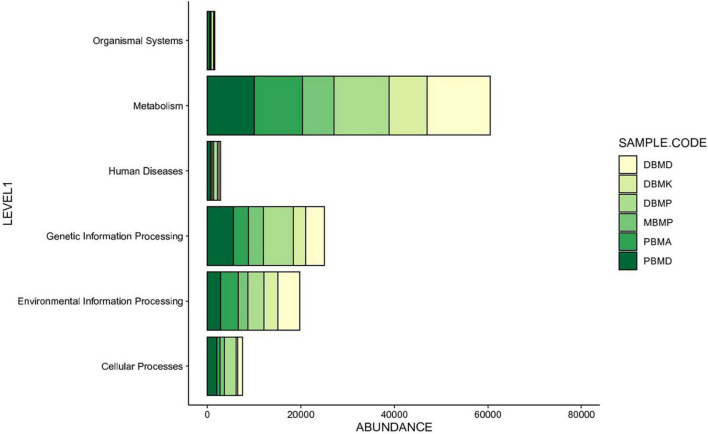
KEGG (level 1) showing the highest number of genes annotated to pathways.

**FIGURE 9:**
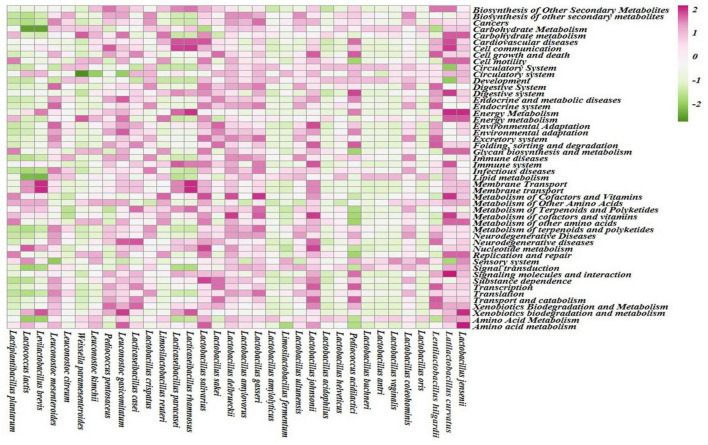
Spearman RS correlation of major bacterial species with KEGG (level 2) pathways.

Different enhanced functional pathways were observed after mapping metagenomic ORFs against eggNOG and KEGG databases. The number of prodigal ORFs identified from *moiya pangsung, moiya koshak, mileye amileye*, and *midukeye* samples were 23,615 (DBMD), 14,447 (DBMK), 25,596 (DBMP), 13,483 (MBMP), 18,329 (PBMA), and 21,710 (PBMD), respectively. About 49.65% of the total mapped ORFs were assigned to the COG functional genes, 49.82% were assigned to the KEGG functional pathways, and the rest 0.53% were assigned to the NOG pathway. In this study, we focused more on the KEGG functional pathways, COG mapping, and NOG pathways. The functional pathways were categorized into three sub-classes *via* KEGG mapping. In level 1, metabolism was the most abundant category followed by genetic information processing, environmental information processing, cellular process, human disease, and organismal system (Figure 8). At level 2, 50 super-pathways, and at level 3, 238 sub-pathways (terminal) from different categories were identified (Supplementary Table 3). Spearman’s rank correlation was performed between some major microorganisms in *moiya pangsung, moiya koshak, mileye amileye*, and *midukeye* samples and different major level 2 super-pathways obtained from KO annotation. *Lactiplantibacillus plantarum, Latilactobacillus curvatus*, and *Lentilactobacillus hilgardii* showed a positive correlation with amino acid metabolism. While, *Lactobacillus jensenii*, and *Lacticaseibacillus casei* showed a positive correlation with lipid metabolism. *Latilactobacillus curvatus* showed the highest correlation with the membrane transport system (Figure 9).

One sample *T*-test was performed to check the intra-sample and inter-sample significance in terms of functional profiles of *moiya pangsung, moiya koshak, mileye amileye*, and *midukeye* samples metagenome. No intra-sample significance was found in any of the pooled samples in terms of functional profiles. The *p-value* for six samples were 0.98 (DBMD), 0.98 (DBMK), 1 (DBMP), 1 (MBMP), 0.99 (PBMA), and 0.99 (PBMA), respectively.

In total, 15 pathways for amino acid metabolism were visualized and annotated to microbial functional genes in the few microbial species in all samples. The most abundant amino acid metabolic pathway visualized in all the fermented bamboo shoot samples was Alanine, aspartate, and glutamate metabolism; ko00250 (16.98%), the next most abundant was glycine, serine, and threonine metabolism; ko00260 (15.97%) as well as cysteine and methionine metabolism; ko00270 (14.97%).

The second most abundant pathway found in all the fermented bamboo shoot samples related to the impartment of flavor and aroma were glycerolipid metabolism (ko00561) (25.33%), fatty acid biosynthesis (ko00061) (25.22%), and glycerophospholipid metabolism (ko00564) (24.02%) (Table 2). A few pathways like the metabolism of terpenoids and polyketides were related to the color and aroma of the fermented bamboo shoot such as carotenoid biosynthesis (ko00906). Pathways of secondary metabolism related to the impartment of flavor and aroma to the fermented bamboo shoots were also annotated such as phenylpropanoid biosynthesis (ko00940), flavonoid biosynthesis (ko00941), flavone and flavonol biosynthesis (ko00944), stilbenoid, diarylheptanoid and gingerol biosynthesis (ko00945), and tropane, piperidine, and pyridine alkaloid biosynthesis (ko00960), etc. Some metabolic pathway related to pectin degradation were identified within carbohydrate metabolisms such as pentose and glucuronate interconversions (ko00040). Pathways related to vitamins metabolism were also annotated such as thiamine metabolism (ko00730), riboflavin metabolism (ko00740), retinol biosynthesis (ko00830), nicotinate and nicotinamide metabolism (ko00760), pantothenate and CoA biosynthesis (ko00770), biotin biosynthesis (ko00780), folate biosynthesis (ko00790), and lipoic acid biosynthesis (ko00785).

**TABLE 2:** KEGG metabolic pathways annotated to different microbial species.

Ko number	KEGG pathway	Microorganism annotated to KEGG pathway
Ko00250	Alanine, aspartate, and glutamate metabolism	*Lactiplantibacillus plantarum, Lactiplantibacillus pentosus, Pediococcus pentosaceus, Lactococcus Lactis, Lactiplantibacillus buchneri, Levilactobacillus brevis, Weissella paramesenteroides*
Ko00270	Cysteine and methionine metabolism	*Lactiplantibacillus plantarum, Lactiplantibacillus fermentum, Levilactobacillus brevis, Weissella paramesenteroides*
Ko00260	Glycine, serine, and threonine metabolism	*Lactiplantibacillus amylolyticus, Lacticaseibacillus casei, Lactiplantibacillus buchneri, Lactiplantibacillus plantarum, Lactiplantibacillus fermentum, Lactiplantibacillus vaginalis Levilactobacillus brevis, Weissella paramesenteroides*
Ko00300	Lysine biosynthesis	*Lactiplantibacillus amylolyticus, Lactiplantibacillus casei, Lactiplantibacillus buchneri, Lactiplantibacillus plantarum, Lactiplantibacillus fermentum, Lactiplantibacillus vaginalis, Levilactobacillus brevis, Weissella paramesenteroides*
Ko00330	Arginine and proline metabolism	*Pediococcus pentosaceus, Lactiplantibacillus plantarum, Lactiplantibacillus brevis, Lactococcus lactis*
Ko00350	Tyrosine metabolism	*Lactiplantibacillus buchneri, Lactiplantibacillus amylolyticus, Lactiplantibacillus plantarum, Lactiplantibacillus pentosus, Lactiplantibacillus hilgardii, Levilactobacillus brevis, Weissella paramesenteroides*
Ko00340	Histidine metabolism	*Lactiplantibacillus buchneri, Lactiplantibacillus plantarum, Lactiplantibacillus fermentum*,
Ko00290	Valine, leucine, and isoleucine biosynthesis	*Lactiplantibacillus pentosus, Lactiplantibacillus casei, Lactiplantibacillus buchneri, Lactiplantibacillus plantarum, Levilactobacillus brevis, Weissella paramesenteroides*
Ko00280	Valine, leucine, and isoleucine degradation	*Lactiplantibacillus buchneri, Lactiplantibacillus amylolyticus, Lactiplantibacillus plantarum, Lactiplantibacillus pentosus, Levilactobacillus brevis, Weissella paramesenteroides*
Ko00360	Phenylalanine metabolism	*Lactiplantibacillus buchneri, Lactiplantibacillus amylolyticus, Lactiplantibacillus plantarum, Lactiplantibacillus pentosus, Levilactobacillus brevis, Weissella paramesenteroides*
Ko00380	Tryptophan metabolism	*Lactiplantibacillus buchneri, Lactiplantibacillus amylolyticus, Lactiplantibacillus plantarum, Lactiplantibacillus pentosus, Lactiplantibacillus hilgardii, Levilactobacillus brevis, Weissella paramesenteroides*
Ko00400	Phenylalanine, tyrosine and tryptophan biosynthesis	*Lactiplantibacillus buchneri, Lactiplantibacillus amylolyticus, Lactiplantibacillus plantarum, Lactiplantibacillus pentosus*

Comparing the dataset of our samples with the NOG database, it was found that all the samples had the maximum number of amino acid transport metabolism unigenes, followed by cell wall/membrane/envelope biogenesis, transcription, and carbohydrate transport metabolism unigenes, respectively (Supplementary Figure 5). COG dataset yielded 97,635 unigenes, from which carbohydrate transport metabolism had the highest number of unigenes in all samples, followed by amino acid transport metabolism and replication, recombination, and repair, respectively. The sample bearing code DBMD has the highest number of unigenes as seen in the COG database than DBMK, PBMA, PBMD, MBMP, and DBMP, respectively (Supplementary Figure 6).

In our bamboo shoot samples, according to the KEGG pathway, there were 80 unigenes for cyano amino acid metabolism among which 46 unigenes from *moiya pangsung*, 3 unigenes *moiya koshak*, 1 from *mileye amileye* and 30 unigenes belonged to *midukeye*. Cyanogenic glycoside can be hypothesized to be metabolized by the cyano amino metabolic pathway *via* tyrosine metabolism.

## 4. Discussion

*Moiya pangsung, moiya koshak, mileye amileye*, and *midukeye* are one of the oldest fermented bamboo shoot gastronomies of Tripura and are prepared by natural fermentation (Uchoi et al., 2015). Profiling of microbial community in naturally fermented *moiya pangsung, moiya koshak, mileye amileye*, and *midukeye* by the shotgun metagenomic method has not been reported yet. Hence, we applied the shotgun metagenomic method to profile the whole microbial community structure in naturally fermented *moiya pangsung, moiya koshak, mileye amileye*, and *midukeye.* In our study, we collected samples from three places in duplicate; *mileye amileye* and *midukeye* were collected from Panisagar and Dharmanagar, *moiya pangsung* was collected from Manubazzar and Dharmanagar, and *moiya koshak* was collected from Dharmanagar. The shotgun sequencing was performed after pooling the DNA of the samples which were all collected in duplicates, because the pooled sequence can also represent the actual sequences from the pooled DNA of the individual samples (Anand et al., 2016) to maintain adequate diversity within and between the samples. We anticipated the microbial community structure to vary within a single representative pool because these samples are naturally fermented. Nucleotide differences and the neutrality test were used to interpret the pooled sample using statistics, and the results clearly showed that there was little polymorphism and variation within the pooled sample, indicating that samples from the same origin have very little diversity differences (Hivert et al., 2018). Negative FST values are also unequivocal proof that there are more alleles than necessary in a single pooled sample, which is truly more than one sample (de Meeûs, 2018). These two tests demonstrate that each of our pooled samples truly has combined data from multiple samples and that there is very little intra-sample variations in the pooled sample, which can also support our pooling method.

Firmicutes was the most abundant phylum in *moiya pangsung, moiya koshak, mileye amileye*, and *midukeye*, like in other Asian fermented bamboo shoot foods like *suansun* of China (Hu et al., 2021), and *tuaithar* of Mizoram, India (Deka et al., 2021). *Lactobacillaceae* was the abundant family in *moiya pangsung, moiya koshak, mileye amileye*, and *midukeye* as reported earlier in other ethnic bamboo shoot fermented foods (Ghosh et al., 2019). *Lactiplantibacillus plantarum* was the most abundant species in all the samples followed by *Lactococcus lactis, Levilactobacillus brevis*, and *Leuconostoc mesenteroides*, which was consistent with other plant-based fermented foods (Lappe-Oliveras et al., 2022) *L. plantarum* attracted many researchers because of its wide applications in the medical field with antioxidant, anticancer, anti-inflammatory, antiproliferative, anti-obesity, and antidiabetic properties (Arasu et al., 2016). *Lactococcus lactis* has been found to be major bacterial species to be found in different fermented bamboo shoot foods as well as other fermented plant-based products (Swain et al., 2014). *Lacticaseibacillus brevis* has been seen to have probiotic and antifungal attributes (Somashekaraiah et al., 2021). *Pediococcus pentosaceus* which has been found in all the samples of the fermented bamboo shoots has been seen to pose a partial characterization of heat-stable, antilisterial, and cell lytic bacteriocin (Halami et al., 2011). Though in a minor abundance, the detection of amino acid-producing bacteria *Corynebacterium glutamicum* (Hahne et al., 2018) and *Bacillus thuringiensis* (Hibi et al., 2011) is an indication of nutritive significance in *moiya pangsung, moiya koshak, mileye amileye*, and *midukeye.*

Though the cumulative abundance of lactic acid bacterial species was > 1‘%, while other genera at lesser abundance than 1% were also detected such as *Bacillus, Cronobacter, Riemerella, Serratia, Yersinia, Clostridium, Stenotrophomonas, Pseudomonas, Listeria, Burkholderia, Oenococcus*, and *Xanthomonas* in all the samples. After bacteria, eukaryota was the most abundant domain found in the metagenome analysis of *moiya pangsung, moiya koshak, mileye amileye*, and *midukeye*. The most abundant eukaryota found in the samples were Ascomycota, Basidiomycota, and Glomeromycota in all the fermented bamboo shoot samples which is consistent with other samples like *Suansun* from China, *tuaithar* from Mizoram, India (Deka et al., 2021), fermented shrimp and sauerkraut (Park et al., 2020). We observed a co-existence of *Lactobacillus* and *Saccharomyces* in all the fermented bamboo shoot samples. Various *non-Saccharomyces* yeasts (*Torulaspora, Lachancea*, and *Starmerella*) were also found. These yeasts are responsible for the addition of aroma (Djeni et al., 2020), glycerol concentration, volatile compounds, and for increasing the concentration of terpenes in fermented foods (Das and Tamang, 2021). Acinetobacter phages were more abundant than other phages in *moiya pangsung, moiya koshak, mileye amileye*, and *midukeye* metagenome. Bacteriophages generally play a critical role in determining bacterial abundance over time (Shapiro et al., 2010). In our study, the abundance of *lactobacillus phages* was found much lower than the abundance of *Lactobacillus* species. However, the presence of phages against the hosts like *Staphylococcus* sp*., Salmonella* sp., and *Streptococcus* sp., indicates the bio-control activity of bacteriophages in food fermentation (Kumar et al., 2019).

Few species of haloarchaea were detected in *moiya pangsung, moiya koshak, mileye amileye*, and *midukeye*, though their abundance was very low. The exact role of archaea in *moiya pangsung, moiya koshak, mileye amileye*, and *midukeye* is still unknown. Halophilic archaea have been reported as the producer of halocin, a compound that infers anti-microbial activity (Corral et al., 2020). Hence the presence of haloarchaea again may be linked with the bio-enhancement of the fermented bamboo shoot food (Mota de Carvalho et al., 2018). Low abundances of the domain eukarya, which included yeasts, filamentous moulds, several species of algae, protozoa, and parasites, were observed in *moiya pangsung, moiya koshak, mileye amileye*, and *midukeye*. Protozoa and parasites are among the other microeukaryotes that are believed to have a detrimental effect on consumers’ health and wellbeing, albeit a comprehensive safety assessment of fermented bamboo shoot cuisine has not yet been conducted. A robust approach that can identify the whole microbial population in a sample with both high and low abundances is the shotgun metagenome sequence (Kumar et al., 2022), may help to understand the safety measure of foods on the basis of their abundances level.

The KEGG annotation showed that metabolic pathways were common in *moiya pangsung, moiya koshak, mileye amileye*, and *midukeye*. One of the predominant predictive functional super-pathways within the metabolism category was amino acid metabolism. During fermentation, alanine, aspartate, and glutamate metabolism and biosynthesis increase the creation of flavor and aroma compounds (Sulaiman et al., 2014). The abundance of genes involved in the synthesis of branched-chain amino acids (valine, leucine, and isoleucine) was also detected from the metagenome analysis of *moiya pangsung, moiya koshak, mileye amileye*, and *midukeye*, which are involved in energy metabolism of *Lactobacillus* sp. (Neinast et al., 2019). The functional study of the *moiya pangsung, moiya koshak, mileye amileye*, and *midukeye* metagenome was used to predict the synthesis and metabolism of glutathione. The cellular processes of anti-oxidant defense, drug detoxification, and cell signaling involved in the regulation of gene expression and apoptosis all involve the metabolism, production, and transport of glutathione (Kennedy et al., 2020). Additionally, computational methods were used to predict the presence of the taurine and hypotaurine production genes, which have been linked to a reduced risk of cardiovascular disease (Xu et al., 2008). According to the COG database, carbohydrate metabolism, RNA processing and modification, chromatin structure and dynamics and cytoskeleton were predominant in all the samples when correlated between COG data and major species which is consistent with the results obtained by Hu et al. (2021). KEGG pathway annotation of samples revealed the catabolism of cyanogenic glycosides, viz., dhurrin, and linamarin through the cyano amino metabolic pathway. It is possible that taxiphyllin may follow the same catabolic process thereby reducing its amount in the fermented bamboo shoots (Appenteng et al., 2021). Terpenoids are also known to be the aroma compound in wine fermentation (Belda et al., 2017). The presence of flavonoids and triterpenes in fermented bamboo shoots has been documented by Lin et al. (2018) to impart flavor to the bamboo shoots. The genes for flavonoid metabolism were also detected from our samples through the culture-independent method. The presence of polygalacturonase enzyme which is referred to as the key enzyme in the pectin degradation pathway may suggest or predict that the bacteria present in fermented bamboo shoots have pectinase degrading ability and soften the texture of the fermented bamboo shoots (Buergy et al., 2020).

The presence of the thiamine metabolic pathway not only indicates that thiamine acts like a coenzyme for various enzymes but also aids in the production of more flavoring substances as opposed to off-flavoring substances (Labuschagne and Divol, 2021). Lactic acid bacteria were the dominant microorganism in the fermented bamboo shoots and previously have been seen to produce riboflavin which is a strong candidate for food biofortification, besides its role in red blood cell production and also aids in the release of energy from proteins (Spacova et al., 2022). The vitamin A component retinol has become an increasingly sought-after cosmetic ingredient (Hu et al., 2022). The presence of a nicotinamide biosynthesis pathway ensures the good absorption of tryptophan and niacin when fermented food is consumed (Faivre et al., 2021). Pantothenate and biotin metabolic pathway help in the growth of various lactic acid bacteria (Yao et al., 2018). The water-soluble vitamin B group member folate, particularly in the forms of tetrahydrofolate (THF) and methyl-tetrahydrofolate (MTHF), is crucial for the carbon-1 transfer processes required for the creation of DNA, nucleic acids, amino acids, and other vitamins (Mahara et al., 2021). These metabolic pathways are attributed to microorganisms fermenting the food, lactic acid bacteria have been found to possess the above KEGG metabolic pathways as previously described in the correlation heat map of major lactic acid bacteria species and KEGG pathways (Cui et al., 2023).

This present study may also provide theoretical and technical support for search and application of the key enzymes produced by the fermenting microbes, and various essential amino acids with health benefits predicted in the fermentation of *moiya pangsung, moiya koshak, mileye amileye*, and *midukeye* for other Asian fermented bamboo shoot. The metataxonomic and predictive functional features of the *moiya pangsung, moiya koshak, mileye amileye*, and *midukeye* metagenome may be used to design the starter culture with enhanced nutritional quality under controlled and hygienic fermentation conditions.

## 5. Conclusion

*Moiya pangsung, moiya koshak, mileye amileye*, and *midukeye* is a delicacy of the Tripura ethnic tribe’s diets and presents one of the most consumed foods of Tripura. There have been no studies on microbiology and nutritional aspects of *moiya pangsung, moiya koshak, mileye amileye*, and *midukeye*. Our study revealed different beneficial microorganisms associated with *moiya pangsung, moiya koshak, mileye amileye*, and *midukeye* fermentation along with their beneficial predictive functional profiles. We hope that the information obtained from this study may help the commercial producers and consumers to be aware of the functional microbial community, the health benefits, and importance of hygiene and food safety during preparation and handling of *moiya pangsung, moiya koshak, mileye amileye*, and *midukeye*. We believe this is the first report on the shotgun-based metataxonomic profile of naturally fermented *moiya pangsung, moiya koshak, mileye amileye*, and *midukeye*.

## Data availability statement

The datasets presented in this study can be found in online repositories. The names of the repository/repositories and accession number(s) can be found in the article/Supplementary material.

## Author contributions

RD carried out sampling, investigation, data curation, formal analysis, and preparation of original draft. BT conceptualized the work and helped in writing and supervision. IN helped in data analysis. NT helped in writing and editing. KM helped in sampling. All authors contributed to the article and approved the submitted version.

## Footnotes

1
https://usegalaxy.org/

## Supplementary material

Supplementary Figure 1A Preparation of *moiya pangsung* a traditional fermented bamboo shoot of Tripura **(a)** tender shoots of *Melanconna baccifera*, **(b)** cutting, **(c)** top portion of the bamboo shoot, and **(d)** soaked in water for fermentation.

Supplementary Figure 1B Preparation of *mileye amileye* a traditional fermented bamboo shoot of Tripura **(a)** tender shoots of *Melanconna baccifera*, **(b)** cutting, **(c)** middle portion of the bamboo shoot, **(d)** soaked in water for fermentation, and **(e)** fermented product.

Supplementary Figure 1C Preparation of *midukeye*, and *moiya koshak* a traditional fermented bamboo shoot of Tripura **(a)** tender shoots of *Melanconna baccifera*, **(b)** cutting, **(c)** middle portion of the bamboo shoot, **(d)** washed with water, **(e)** wrapped in banana leaves for fermentation, and **(f)** fermented product.

Supplementary Figure 2 Heat map representation of major species in *moiya pangsung, mileye amileye, moiya koshak*, and *midukeye*.

Supplementary Figure 3A Stacked bar plot representation of major fungal phylum communities in *moiya pangsung, mileye amileye, moiya koshak*, and *midukeye*.

Supplementary Figure 3B Heat map representation of major fungal family communities in *moiya pangsung, mileye amileye, moiya koshak*, and *midukeye.*

Supplementary Figure 3C Heat map representation of the major fungal species communities in *moiya pangsung, mileye amileye, moiya koshak*, and *midukeye*.

Supplementary Figure 4 The shared and unique species were visualized between samples using a venn diagram.

Supplementary Figure 5 NOG pathways annotated.

Supplementary Figure 6 COG pathways annotated.

Supplementary Table 1 Fermented bamboo shoots samples collected from different locations in Tripura, India.

Supplementary Table 2 Abundance of bacteria at family level in *moiya pangsung, mileye amileye, moiya koshak*, and *midukeye*.

Supplementary Table 3 KEGG gene abundance in each sample in levels 2 and 3.
