# Supplementary figures and images for: First report on metagenomics and their predictive functional analysis of fermented bamboo shoot food of Tripura, North East India
Source: Front Microbiol. 2023 Apr 12;14:1158411. doi: 10.3389/fmicb.2023.1158411 (PMC10130461; doi:10.3389/fmicb.2023.1158411)

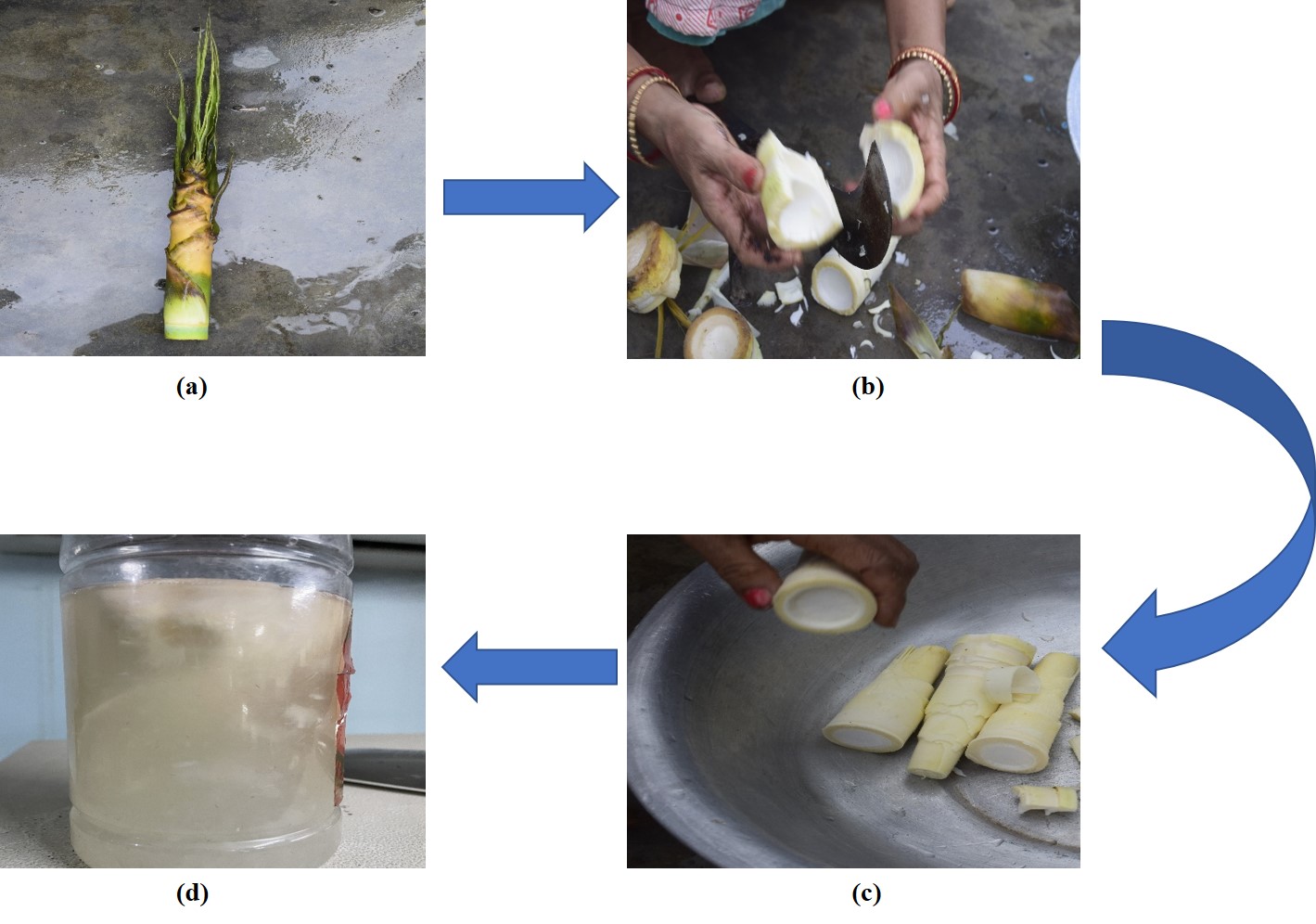

Supplement: Supplementary Figure 1A — Preparation of moiya pangsung a traditional fermented bamboo shoot of Tripura (a) tender shoots of Melanconna baccifera, (b) cutting, (c) top portion of the bamboo shoot, and (d) soaked in water for fermentation. [file Image_1.JPEG]

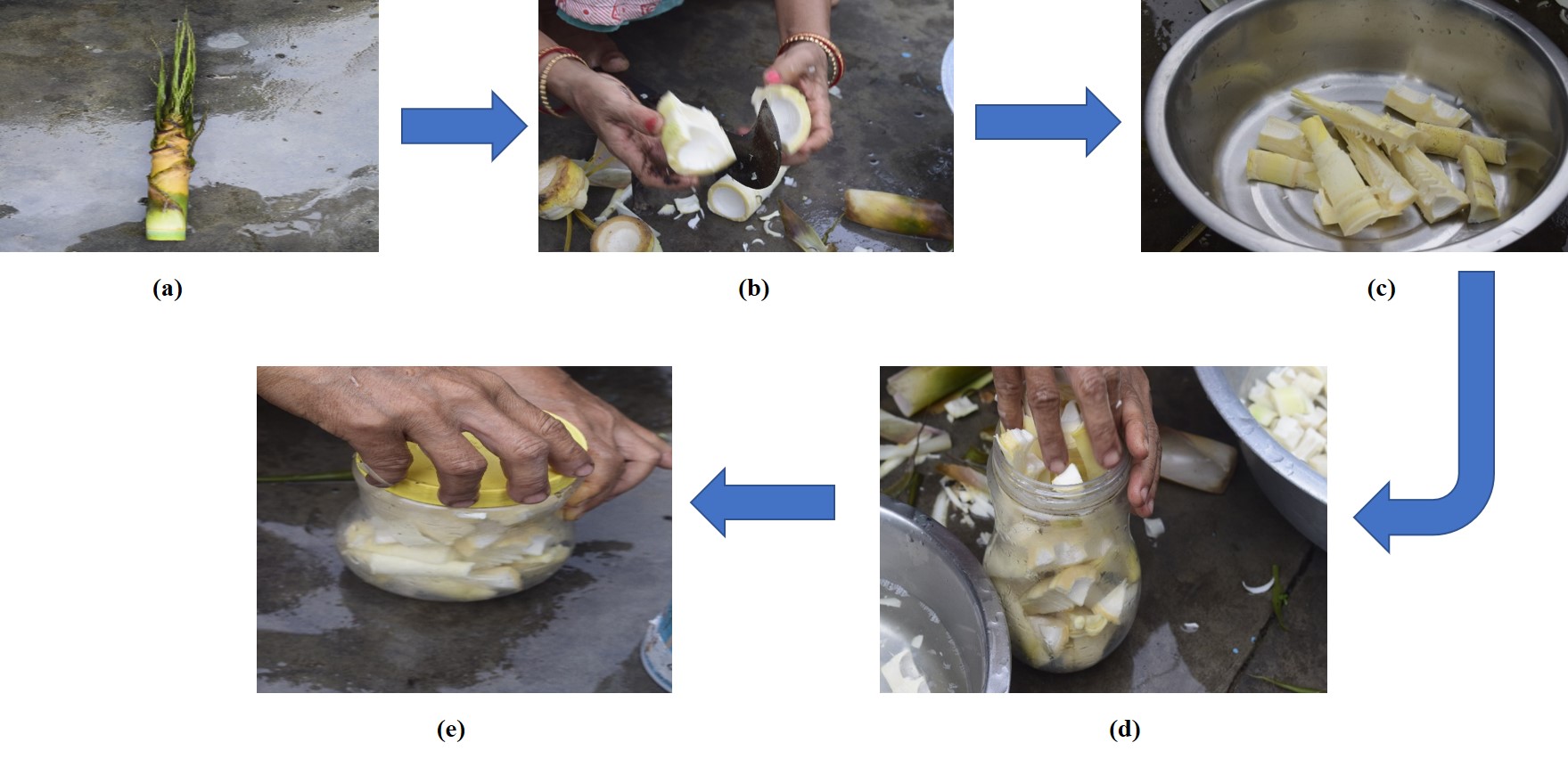

Supplement: Supplementary Figure 1B — Preparation of mileye amileye a traditional fermented bamboo shoot of Tripura (a) tender shoots of Melanconna baccifera, (b) cutting, (c) middle portion of the bamboo shoot, (d) soaked in water for fermentation, and (e) fermented product. [file Image_2.JPEG]

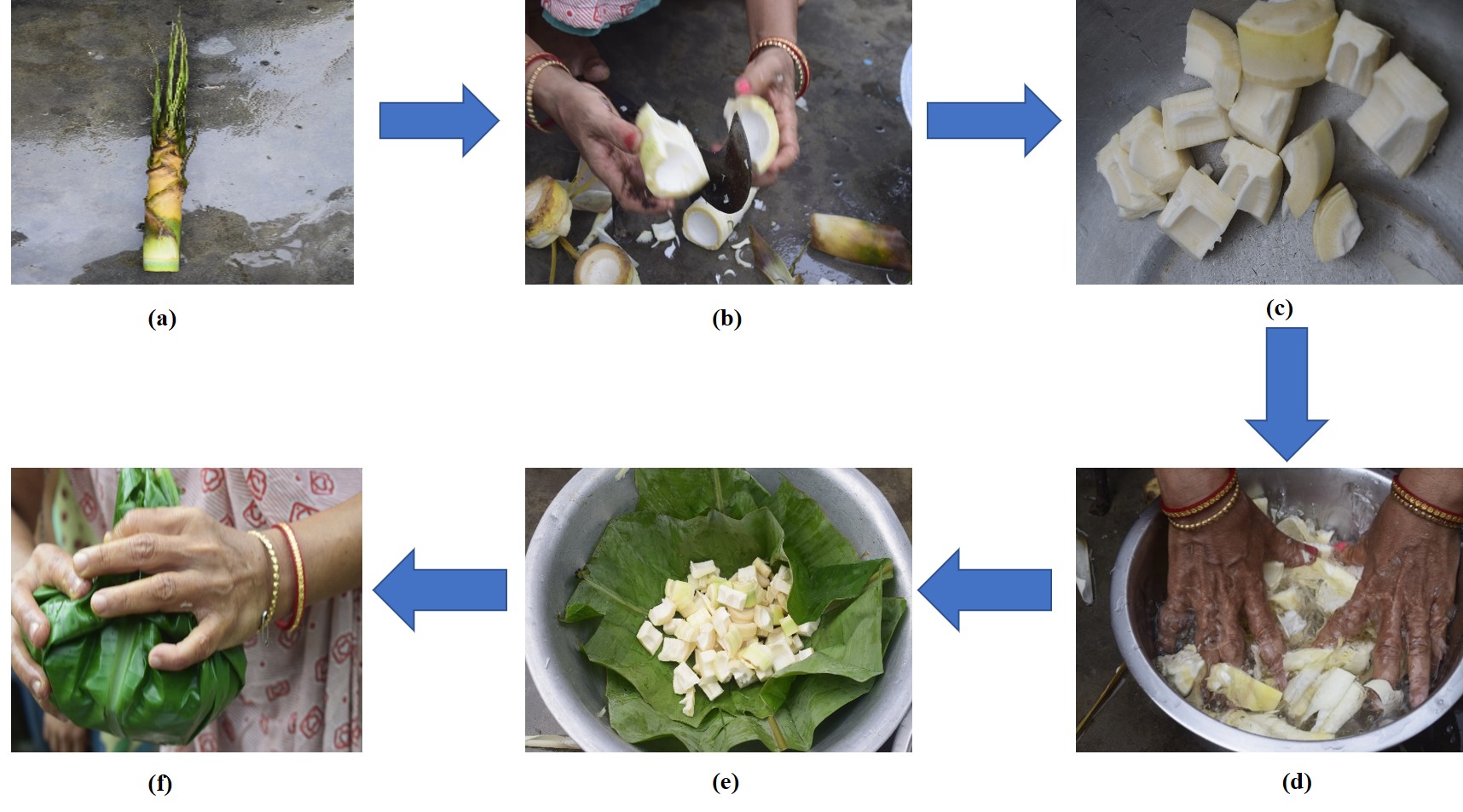

Supplement: Supplementary Figure 1C — Preparation of midukeye, and moiya koshak a traditional fermented bamboo shoot of Tripura (a) tender shoots of Melanconna baccifera, (b) cutting, (c) middle portion of the bamboo shoot, (d) washed with water, (e) wrapped in banana leaves for fermentation, and (f) fermented product. [file Image_3.JPEG]

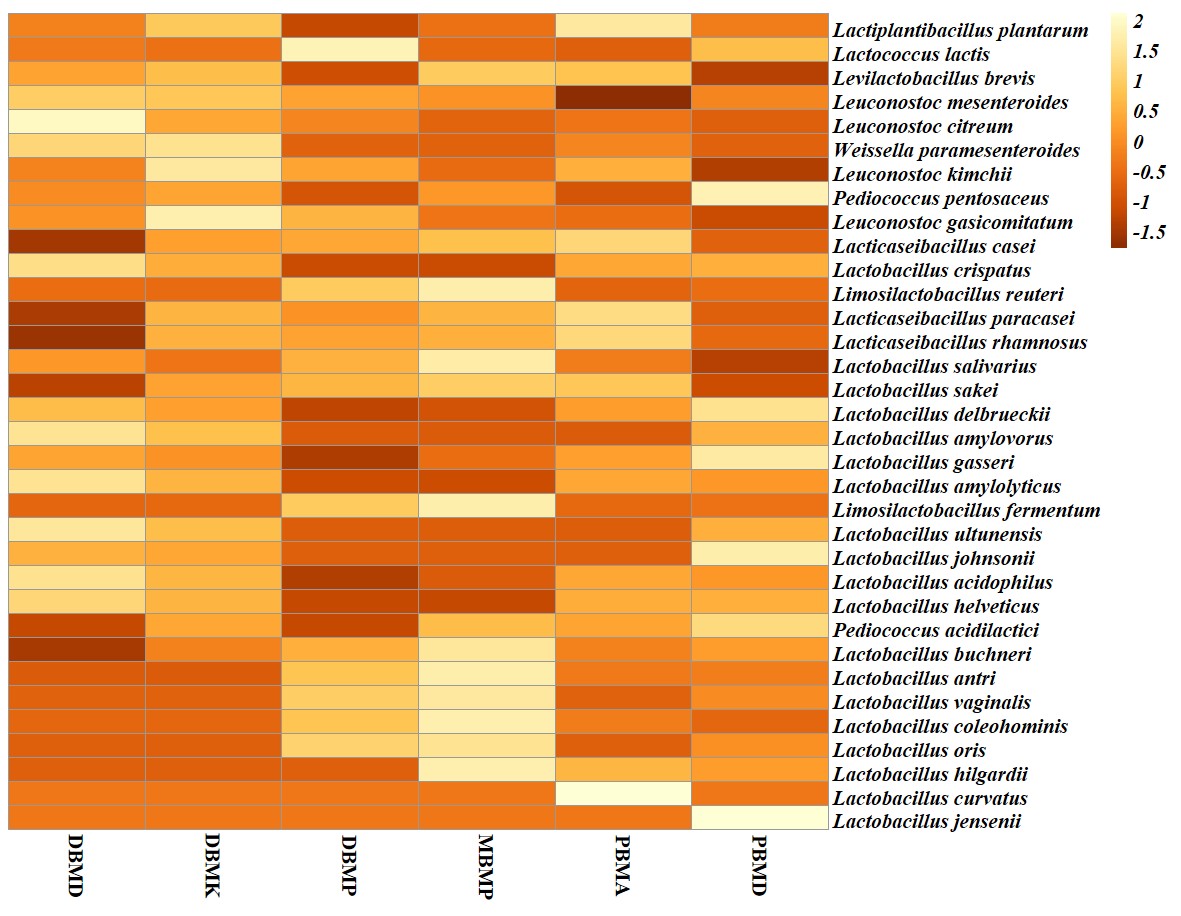

Supplement: Supplementary Figure 2 — Heat map representation of major species in moiya pangsung, mileye amileye, moiya koshak, and midukeye. [file Image_4.JPEG]

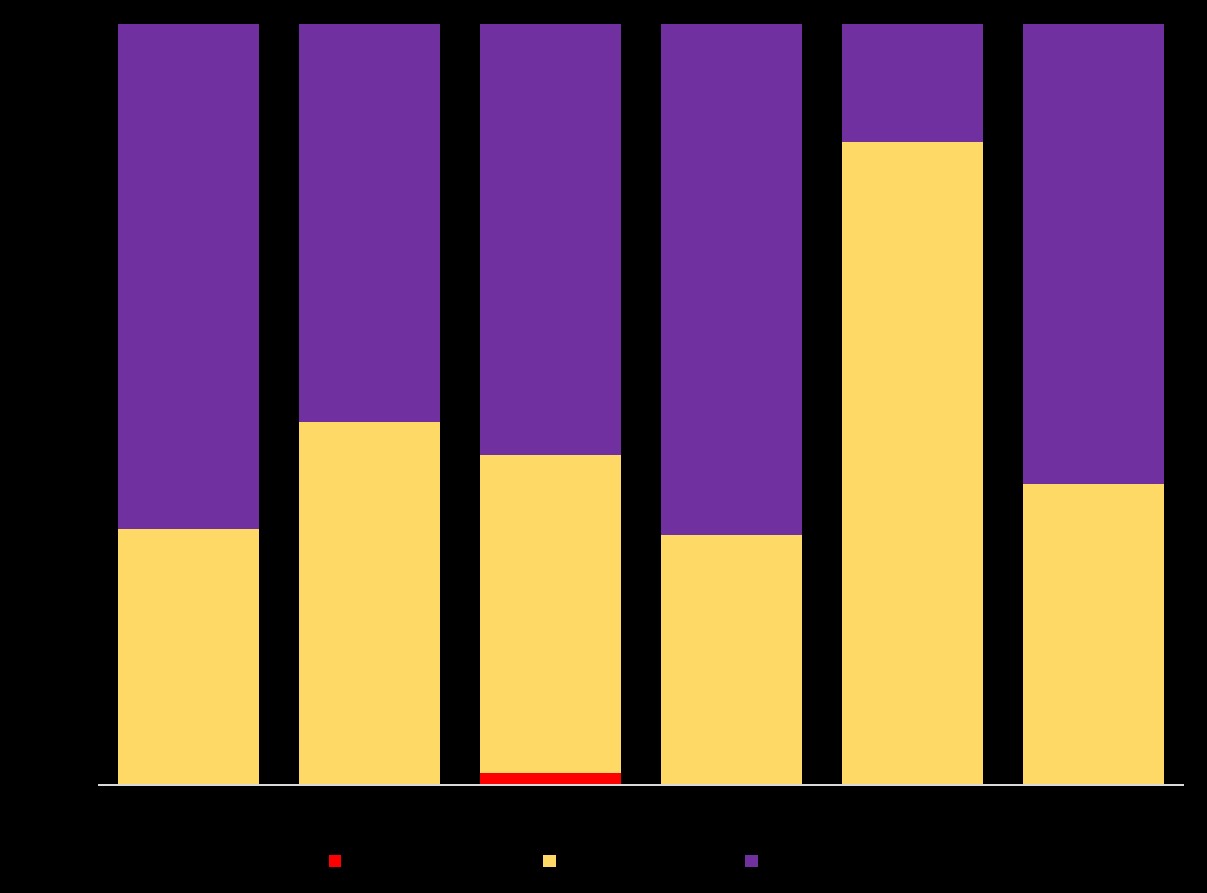

Supplement: Supplementary Figure 3A — Stacked bar plot representation of major fungal phylum communities in moiya pangsung, mileye amileye, moiya koshak, and midukeye. [file Image_5.JPEG]

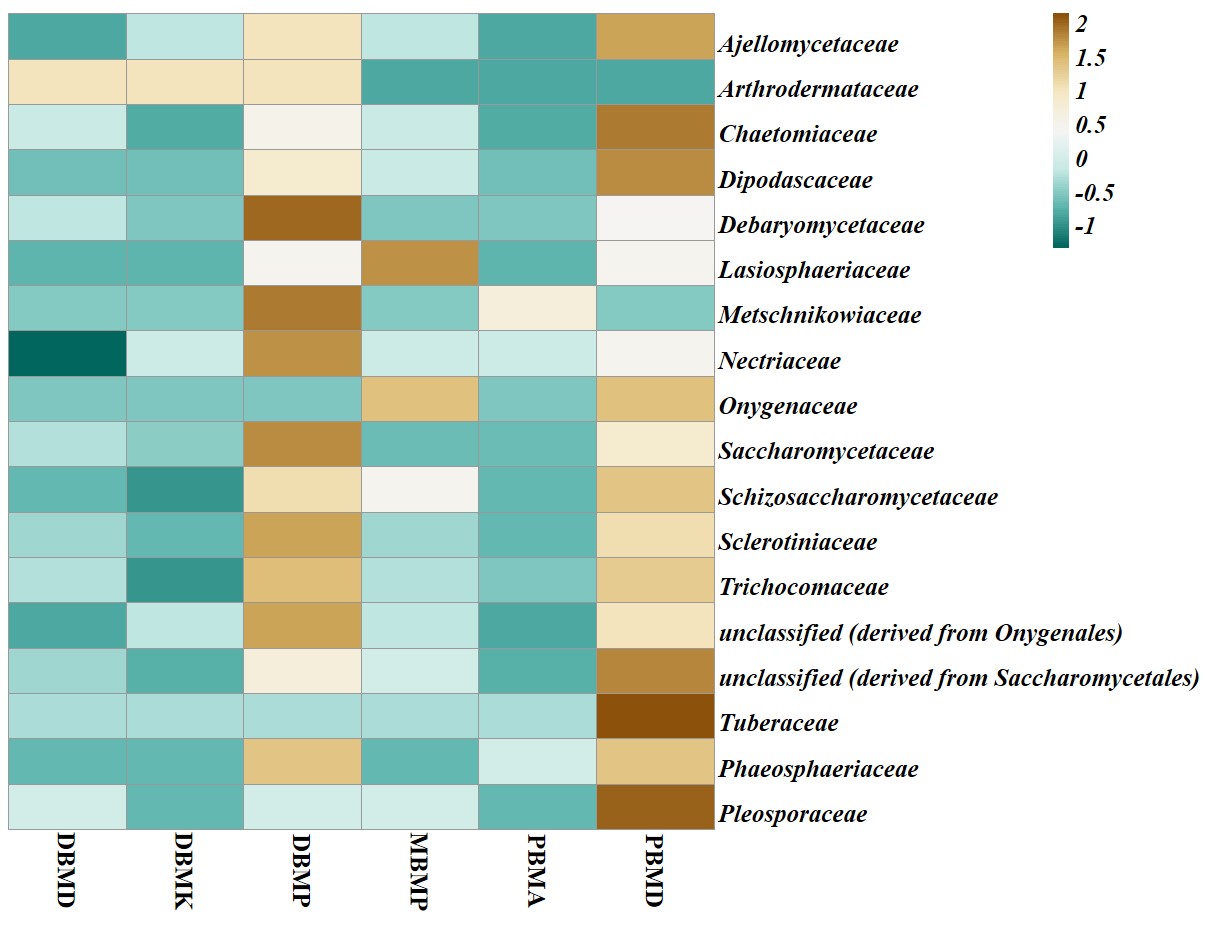

Supplement: Supplementary Figure 3B — Heat map representation of major fungal family communities in moiya pangsung, mileye amileye, moiya koshak, and midukeye. [file Image_6.JPEG]

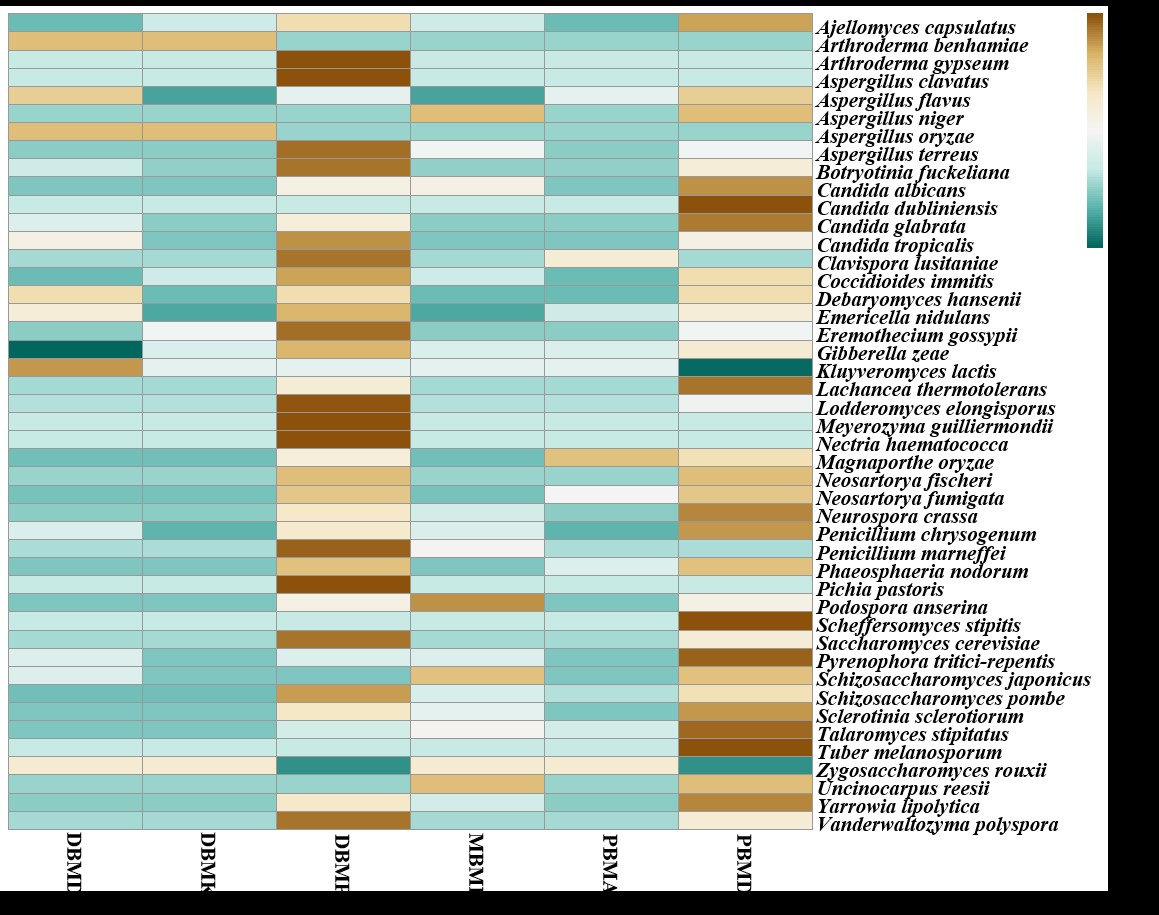

Supplement: Supplementary Figure 3C — Heat map representation of the major fungal species communities in moiya pangsung, mileye amileye, moiya koshak, and midukeye. [file Image_7.JPEG]

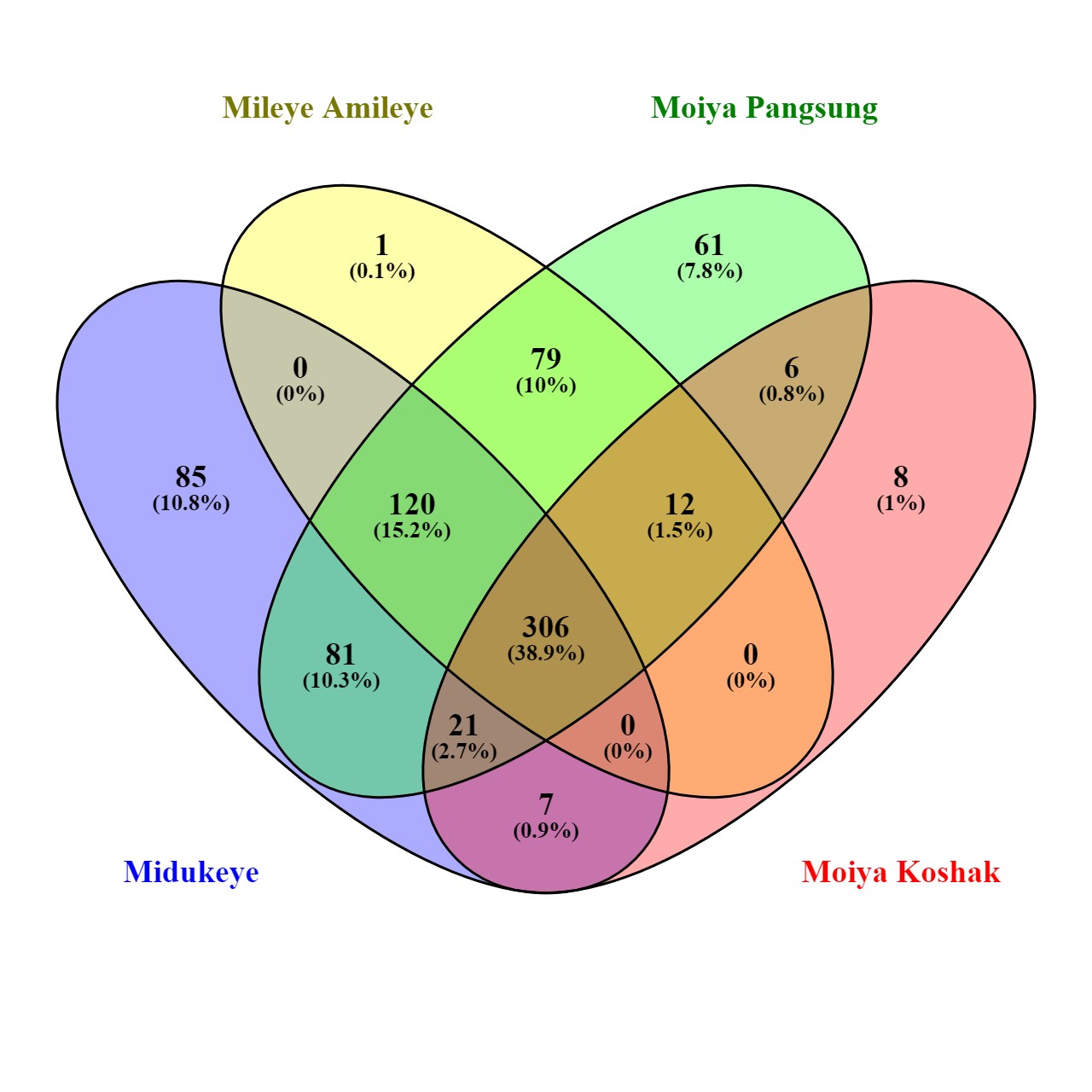

Supplement: Supplementary Figure 4 — The shared and unique species were visualized between samples using a venn diagram. [file Image_8.JPEG]

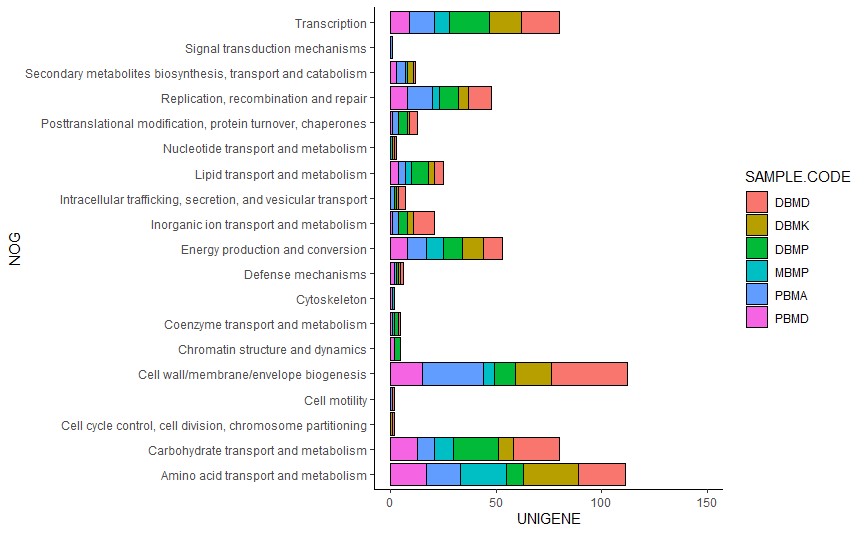

Supplement: Supplementary Figure 5 — NOG pathways annotated. [file Image_9.JPEG]

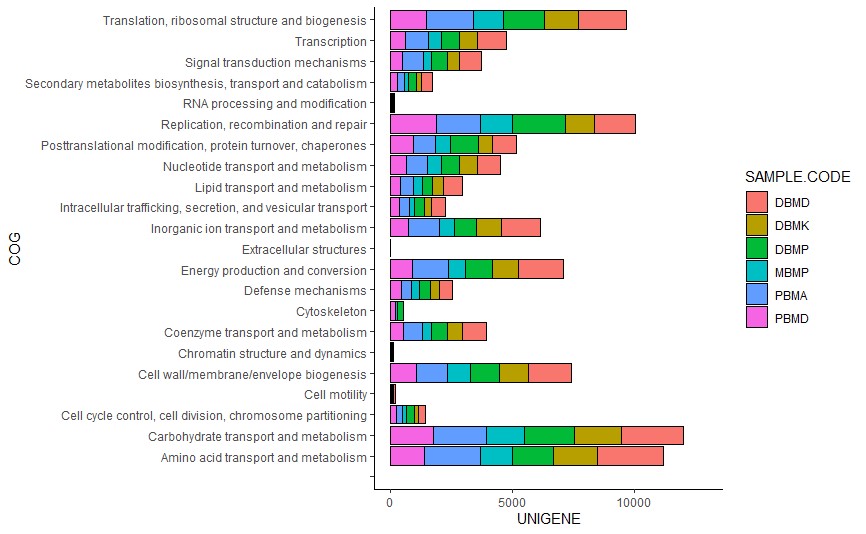

Supplement: Supplementary Figure 6 — COG pathways annotated. [file Image_10.JPEG]
