# Supplementary material for: First report on metagenomics and their predictive functional analysis of fermented bamboo shoot food of Tripura, North East India
Source: Front Microbiol. 2023 Apr 12;14:1158411. doi: 10.3389/fmicb.2023.1158411 (PMC10130461; doi:10.3389/fmicb.2023.1158411)
Supplement: Supplementary Table 1 — Fermented bamboo shoots samples collected from different locations in Tripura, India. [file Table_1.docx]

| **Ethnic group** | **Fermented bamboo shoot food** | **Sample type and part of bamboo shoot used** | **Sample collection location** | **Sample code** |
| --- | --- | --- | --- | --- |
| Chakma | Mileye Amileye  Midukeye | semi-dry, middle part | Panisagar  Dharmanagar, Panisagar | PBMA  DBMD  PBMD |
| Uchoi | Moiya Pangsung | Wet and submerged, the top part | Dharmanagar, Manubazzar | DBMP  MBMP |
| Debbarma | Moiya koshak | Wet and submerged, middle part | Dharmanagar | DBMK |
